# Attending veterinarians improve the research capability and psychological well-being of researchers in animal research institutes

**DOI:** 10.3389/fvets.2023.1340225

**Published:** 2024-01-05

**Authors:** Ji Min Lee, Gwang-Hoon Lee, KilSoo Kim

**Affiliations:** ^1^Research Ethics Team, Seoul National University, Seoul, Republic of Korea; ^2^Preclinical Research Center, Daegu-Gyeongbuk Medical Innovation Foundation, Daegu, Republic of Korea; ^3^Department of Veterinary Toxicology, College of Veterinary Medicine, Kyungpook National University, Daegu, Republic of Korea

**Keywords:** attending veterinarians, professional quality of life, animal welfare, care-killing paradox, animal research

## Abstract

The use of laboratory animals in biomedical research has significantly advanced scientific understanding, yet it raises ethical concerns about animal welfare and the mental health of researchers Recent research has highlighted the potential for stress and compassion fatigue among researchers working with distressed animals. Attending veterinarians (AVs) are crucial in mitigating the pain and stress experienced by animals and, by extension, researchers. However, the impact of AVs on researchers’ psychological well-being remains understudied. This study explores how AVs contribute to researchers’ research capability and psychological well-being in animal research institutions. AVs oversee animal housing, health, and welfare; their involvement is mandated or strongly recommended in developed countries. AVs enhance animal welfare by ensuring proper housing, nutrition, and social interaction. They monitor animal health, educate researchers on pain assessment, and promote compliance with post-surgical care. AVs also contribute to researchers’ well-being by addressing euthanasia procedures, which can be emotionally challenging. Programs for rehoming animals after experiments offer an alternative to euthanasia and positively impact researchers’ psychological well-being. Moreover, AVs promote workplace well-being by fostering positive workplace cultures, offering peer counseling, and providing social support. Programs considering animal welfare and researchers’ emotions are crucial for a healthy research environment. In conclusion, AVs are essential in balancing scientific progress with animal welfare and researchers’ psychological well-being. Therefore, their role should be recognized as vital in achieving social equity that considers the welfare of humans and laboratory animals.

## 1 Introduction

Laboratory animals are raised in confined spaces to conduct experiments, including evaluating the efficacy and safety of drug candidates or assessing medical device performance (1–3). Biomedical research using laboratory animals has recently made tremendous progress (4, 5). The quantity of animals employed for educational and research purposes experienced a rise across the country, surging from 2.5 million in 2015 to 4.9 million in 2021 (6). Animal experimentation is essential in the preclinical evaluation of new drugs and medical devices; however, it unavoidably subjects animals to pain and stress (7–9). Increasing societal concern for animal welfare has led to efforts to enhance the well-being of laboratory animals, emphasizing the provision of enriched environments and larger housing spaces that respect their natural behaviors (10–12). Furthermore, stress can induce various physiological changes in animals, potentially impacting the accuracy and reliability of results in non-clinical studies. Therefore, promoting animal welfare measures that reduce stress can enhance the precision and credibility of outcomes in non-clinical research (13–15).

Previous research has primarily focused on alleviating animal stress, confirming their positive effects on laboratory animals (16, 17). However, recognizing the interplay of emotions between humans and animals is essential (18–20). Animal stress and pain can induce severe stress in researchers (18, 19). Furthermore, the negative public perception of animal experimentation and the “care-killing paradox,” where researchers may need to euthanize animals they have cared for, can lead to stress for researchers (21, 22). In March 2020, a research laboratory faced the imperative of euthanizing a substantial number of experimental animals due to the closure of animal facilities prompted by the COVID-19 pandemic. This unforeseen circumstance revealed a paradoxical dilemma, as researchers were compelled to euthanize animals that had been under their care. Reports surfaced of individual researchers having to personally euthanize hundreds of mice daily. They described the emotional toll of this task, expressing feeling psychologically and physically “ethically fatigued” (23). Additionally, instances such as the foot-and-mouth disease disaster have highlighted the psychosocial trauma experienced by individuals involved in the euthanasia of numerous animals, emerging as a societal concern. The impact of these situations extends beyond the immediate ethical considerations to encompass profound emotional and psychological challenges for those tasked with making ethically fraught decisions in the interest of public health and safety (24).

One study has suggested expanding the foundation of animal experimentation, known as the 3Rs (Replacement: maximizing the use of alternative research methods in lieu of animal trials, whenever possible, Reduction: decreasing the quantity of animals sacrificed for research purposes, and Refinement: employing approaches that mitigate the suffering of animals involved in experiments), to include humans and emphasized the importance of using supportive methods to alleviate work-related stress (25, 26).

Stress experienced by animals can be transmitted to researchers, leading to a decrease in their professional quality of life and perpetuating a harmful cycle affecting the animals (27). Researchers working with animals experiencing pain and stress may experience more stress than those in non-laboratory animal facilities (27), possibly causing post-traumatic stress disorder, a psychologically debilitating condition in individuals performing distressing animal experiments (28). Moreover, actions compromising the basic welfare of laboratory animals and inflicting pain are unacceptable to many members of society, potentially undermining public support for animal research. Paradoxically, enhanced status for laboratory animals can become a source of moral stress for researchers (29, 30). Moral stress arises when professional duties conflict with personal ethical convictions or societal moral norms. Individuals often find themselves having to euthanize healthy animals when choosing a profession that revolves around protecting animals and respecting life, countering societal moral perspectives. Therefore, the moral obligation inherent in the job, which entails performing euthanasia on animals or subjecting them to procedures causing distress, can expose researchers to moral stress due to the heightened societal awareness surrounding animal use (31–33).

However, researchers may prioritize their immediate experimental tasks over animal welfare, highlighting the crucial role of the attending veterinarian (AV) in mitigating animal pain and stress. AVs play a pivotal role in the welfare of laboratory animals. The responsibilities of AVs, as specified in the Animal Welfare Act in the United States, encompass overseeing the housing, sustenance, and welfare of research animals, participating as voting members in the Institutional Animal Care and Use Committee (IACUC), ensuring appropriate veterinary services for research animals, and offering guidance to primary investigators and other staff on animal management, immobilization, anesthesia, pain relief, calming, and humane euthanasia practices (34, 35). AVs can also support researchers feeling burdened by causing distress to animals through inexperienced experimental techniques. Additionally, AVs can enhance the welfare of experimental animals and alleviate researchers’ compassion fatigue and stress associated with animal experimentation. The participation of AVs in the review of animal experimental protocols provides researchers with a sense of reassurance regarding implementing experiments applying the 3R principles. This confidence enhances research efficiency, reducing the unnecessary use of laboratory animals.

Efforts are being made to mandate the use of AVs to enhance the welfare of laboratory animals in countries such as the United States and Japan, which have significant interest in animal rights. However, many countries still lack awareness of animal rights and consequently overlook the need for AV employment (1, 36). Furthermore, even in countries emphasizing animal rights, some researchers who neglect animal welfare may encounter difficulties establishing mutual trust with AVs.

Professional compassion fatigue among animal research scientists can lead to reduced empathy, decreased caregiving behavior, increased anger, frustration, alcohol and drug use, impaired professional decision-making, absenteeism, heightened anxiety in society, irrational fears, interpersonal relationship problems, and other issues within the workplace (25). These negative effects can be mitigated through social support. AVs, as one available form of social support, can leverage their veterinary expertise to enhance animal welfare, positively impacting the interaction between animals and researchers, helping overcome professional compassion fatigue and promoting mental well-being (22). However, no research has been done on the impact of AVs on the mental health of researchers. In this study, we discuss the role of AVs in collaborating with researchers to promote animal welfare. This research aims to underscore the significance of AV duties by examining their impact on researchers’ research capabilities and psychological well-being to enhance the overall well-being of individuals engaged in animal research. Through a comprehensive literature review, we endeavored to elucidate the outcomes of prior studies and discern the current trends in the field. Subsequently, we conducted an investigation into the management of laboratory animals and euthanasia practices by interviewing experts in the field, particularly AVs, to gain insights into their on-the-ground experiences. This approach allowed us to integrate scholarly findings with firsthand perspectives from professionals directly involved in the care and ethical considerations surrounding experimental animals.

## 2 Expanding the role of AVs

### 2.1 Global enhancement of animal welfare and the role of AVs

Many countries have recognized the ethical concerns of modern society regarding using animals in research and have developed regulatory frameworks to support the humane management and use of laboratory animals (37). According to a UK opinion poll, 65% of the public accepts the use of animals for medical purposes and 68% for scientific purpose (38). However, this acceptance depends on the research situation and the experimental animal species, and it is possible when alternative methods are non-available or unnecessary pain is avoided. Interest in alternative methods to animal testing also increased from 55 to 60% compared to 2 years ago (38). Public opinion in the UK regarding the use of animals in research indicates that 71% accept experimentation under the condition of no alternative methods and minimal animal suffering. Animal welfare is a key concern, with 35% opposing animals’ use in research on ethical grounds, and 54% expressing a desire for more information on efforts to improve animal welfare in research. Legislation reflects these views, emphasizing the need to minimize both animal use and suffering in scientific endeavors (39). European Union (EU) guidelines, which define minimum standards for animal experimentation implemented by 28 EU member states, specify the need to ensure that “procedures that may cause severe pain, suffering, or distress that is long-lasting and cannot be alleviated are not performed.” DIRECTIVE 2010/63/EU is on the protection of animals used for scientific purpose (refer 2). Recital 23 of the Directive states that “From an ethical standpoint, there should be an upper limit of pain, suffering and distress above which animals should not be subjected in scientific procedures. To that end, the performance of procedures that result in severe pain, suffering or distress, which is likely to be long-lasting and cannot be ameliorated, should be prohibited.” The 27 member states of the EU observe the animal welfare standards of DIRECTIVE 2010/63/EU (40). In the case of laboratory animals, pain can sometimes escalate due to experimental protocols or *in vivo* analyses, as effective measures to eliminate threats to animals cannot always be applied (28). In certain experiments, it may be necessary to induce transient discomfort in animals. For instance, when determining the Approximate Lethal Dose (ALD) or No Observed Adverse Effect Level (NOAEL), there are situations where analgesics cannot be administered, and the administration of substances with specific properties is required to elicit particular reactions. Furthermore, in assessing the safety of medical products, it is unavoidable to induce discomfort. Therefore, strict justifications must be established when inflicting unavoidable suffering on animals (41). Judgment should be made by a specialist with scientific and ethical expertise to achieve this, and well-trained AVs are qualified professionals to provide such assessments. AV should graduate from a veterinary medical college, undergo training and/or gain experience in the care and management of the relevant species. AV must also possess sufficient authority, as provided by the institution, to treat an animal (42, 43). AV is an experienced professional who possesses extensive knowledge in veterinary medicine and understands the biological characteristics of the animal, enabling appropriate clinical treatment (44). Regulations on animal experimentation encompass various aspects, including ethical review bodies and principles, personnel qualifications, facilities, resources, technological procedures, animal care and use, industrial health and safety, transportation, and recommended ethical review processes and standards. Of particular note is the regulation governing AV recruitment (1). AV appointments are mandatory or strongly recommended in countries with high societal demands for animal welfare, such as China, Europe, Israel, South Korea, and United States contrary to India, Indonesia, Japan, Malaysia, Singapore, and Thailand (Table 1) (1, 45, 46).

**Table 1:** Comparison of the act on AVs employment.

	Required	Not required
AVs employment	China, Europe, Israel, South Korea, and United States.	India, Indonesia, Japan, Malaysia, Singapore, and Thailand.

Article 25 of Directive 2010/63/EU requires that ‘each breeder, supplier and user has a designated veterinarian with expertise in laboratory animal medicine, or a suitably qualified expert where more appropriate, charged with advisory duties in relation to the wellbeing and treatment of the animals’ Under Directive, DV involved in activities related to the functions of ethical committees, facility management, health monitoring, research projects, occupational health and safety, compliance with legislation and the education and training of staff and persons using animals for experimental purposes (47, 48). In the United Kingdom, ‘Named Veterinary Surgeons (NVS)’ offers guidance on the well-being of animals housed in scientific facilities. Under the Animal Scientific Procedures Act, the NVS must uphold the principles of the 3Rs, assuming the responsibility of overseeing and advising on the health, welfare, and treatment of animals (49). The AVs system is not legally required in many Asian countries, including, India, Indonesia, Japan, Malaysia, Singapore, and Thailand (1, 50). In China, Europe, Israel, South Korea, and United States., the Animal Welfare Act was amended, requiring organizations engaged in animal testing to employ AVs (40, 42). However, in countries where societal awareness of animal welfare is limited or where understanding of animal welfare for laboratory animals is lacking, a corresponding deficiency in recognizing the role of AVs may exist. Consequently, this AV awareness deficiency can potentially compromise the welfare of laboratory animals.

### 2.2 Animal welfare programs offered by AVs to enhance researchers’ mental health in animal research institutions

In this study, we categorize the role of AVs in enhancing the mental health of researchers into two primary aspects. First, we address the direct promotion of animal welfare by AVs. The goal of enhancing the welfare of laboratory animals is to meet their physiological and behavioral needs appropriately while promoting ethical treatment through adequate housing, nutrition, and social interaction (37, 51, 52). Provision of a minimum living space for each animal species, along with the implementation of appropriate environmental enrichment, is essential. Utilizing social housing whenever possible, instead of single housing, respects the natural tendencies of the animals. Facilitating positive reinforcement training, including cooperative engagement in goal-oriented activities, can enhance management practices and reduce stress (16, 34, 53–55). In a previous study, aggression in pigs decreased following the implementation of social housing and the expansion of floor space in an animal research facility. Additionally, a reduction in plasma cortisol levels, indicative of decreased stress, was reported (56).

Unlike other animals, laboratory animals are confined in limited spaces and often exhibit stereotypic behaviors and distress due to their highly routine daily activities (57). Animal emotions can interact with those of humans when individuals interact with animals daily, potentially elevating human distress levels (58). Individuals responsible for enhancing animal welfare, such as AVs and managers, demonstrated higher empathy and job satisfaction than researchers, whereas Ph.D. students involved in experiments exhibited higher compassion fatigue (22). Furthermore, researchers who implemented environmental enrichment demonstrated a higher professional quality of life (22).

Animal healthcare is a crucial role of AVs in promoting the mental health of researchers during animal experiments. The responsibility of AVs is to monitor the health status of laboratory animals and provide appropriate medical interventions to ensure their well-being (59). Animal pain is primarily expressed through non-verbal means, mainly behavior, and should be evaluated as realistically and objectively as possible. Scientific methods such as grimace scales should accompany behavioral assessments (60, 61). AVs educate researchers on these scales and prescribe appropriate analgesics, leveraging their veterinary knowledge to assess and alleviate pain (59). Non-compliance with post-surgical management and post-surgical analgesia for animals experiencing ongoing pain is a prevalent issue, often promptly rectified through AV involvement. Importantly, procedures should be established within the institution to ensure compliance with post-surgical care.

Regular playtimes for animals, such as dogs and pigs, in playgrounds can serve as environmental enrichment, benefiting the welfare of laboratory animals and the psychological well-being of observing researchers. The provision of engaging activities for animals can contribute to the reduction of stereotypic behaviors and enhance social interactions among them, fostering a positive atmosphere in the experimental environment. Moreover, it can offer researchers the opportunity for direct interaction with animals in a playground setting, promoting mutual. Additionally, routine health check-ups performed on animals, especially larger laboratory animals such as dogs, pigs, and primates, can be perceived as high-quality welfare and provide researchers with psychological reassurance. The maintenance of animals in a healthy state not only enhances the reliability of experimental outcomes but also allows for the minimization of the number of animals used in research. Consequently, meeting ethical responsibilities while reducing the burden on researchers can be achieved.

The Institutional Animal Care and Use Committee (IACUC) plays a dual role in animal experimentation, approving research protocols and supervising post-approval management. AVs can collaborate with the IACUC or serve as members of the IACUC to enhance animal welfare (62, 63). Systems that clandestinely inspect reported harmful animal welfare conditions supplement regular animal welfare checks as part of the Program of Animal Monitoring. Such systems reduce animal distress and stress among researchers, who perceive their workplace as comfortable and psychologically safe.

### 2.3 Direct methods for enhancing the mental health of researchers beyond animal welfare promotion

Secondly, AVs can contribute to improving the mental health of researchers through direct means. One of the most critical aspects in this regard is euthanasia. Euthanasia of laboratory animals involves the deliberate act of causing death to minimize unnecessary pain and stress to animals (64, 65). Unlike companion animals, the euthanasia timing for laboratory animals can be determined from the planning stages of the experiment or anticipated if the animal’s suffering outweighs additional data collection (66). Euthanasia procedures must be conducted carefully to ensure minimal pain and stress, and one of the critical criteria for euthanasia is its psychological impact on researchers (67). Individuals who have witnessed the death of animals, not limited to laboratory animals, may be exposed to excessive stress, substantially impacting their mental health. Many researchers are chronically exposed to “moral stress,” as their typical duties ultimately involve euthanizing animals or engaging in practices that cause pain, suffering, disease, and other harmful conditions required for research (30, 32, 33). According to a study, researchers who have experienced animal stress and pain tend to exhibit higher levels of compassion fatigue, and there is also a reported association between the use of physical euthanasia methods and researcher burnout (22). Individuals working in the field of laboratory animal welfare, such as animal facility staff, demonstrated higher levels of Professional Quality of Life and compassion satisfaction compared to researchers (27). AVs play a mediating role in the management of research and animal interventions, highlighting the need for emotional relief programs for AVs themselves. The AV should ensure that ethical procedures and regulations are adhered to by researchers when performing euthanasia to mitigate their stress. Researchers using physical methods such as cervical dislocation for euthanasia have reported higher burnout levels (22). Therefore, proposing humane methods, such as CO_2_ displacement within 30–70%, to minimize animal distress during euthanasia is essential. Additionally, constructing CO_2_ chambers using non-transparent materials can alleviate visual stress. Furthermore, euthanasia itself and the anticipatory grief that can occur before euthanasia induce extreme stress among researchers. Therefore, AVs should ensure that researchers adhere to ethical procedures and rules when performing euthanasia, thereby reducing the various stresses they may experience, including the care-killing paradox.

Notably, while euthanasia is one of the critical considerations in animal experimentation, it is not an inevitable outcome, and scientific, ethical, and legal frameworks should be carefully contemplated (68). One such means is rehoming. Programs have recently become more active in rehoming animals such as rodents, rabbits, and beagles after completing experiments (69, 70). ‘Rehoming’ is defined as a change in residence for an animal previously used or designated for scientific purposes, where the animal resides for the remainder of its life in a setting appropriate for its requirements without undergoing any additional scientific procedures (69, 71, 72). Directive 2010/63/EU issued by the European Parliament and Council on September 22, 2010, stipulates that upon completing a scientific procedure, the optimal course of action regarding an animal formerly used or designated for scientific purposes should be determined considering animal well-being and potential environmental hazards (40). Rehoming provides researchers with an opportunity to provide animals with a better quality of life without euthanizing them, allowing them to move beyond the distress associated with euthanasia. Rehoming provides a higher level of welfare to laboratory animals and can also boost the morale and well-being of those caring for the animals (73). However, successful rehoming is not guaranteed for all animals. According to one study, 6.2% of rehomed beagles experienced failed adaptation and were returned to research facilities (74). Rehoming efforts should be undertaken following thorough veterinary examinations and socialization training by AVs to enhance the likelihood of successful reintegration into new environments. Since AVs must provide animal welfare and treatment consultation, they should be involved in adoption decisions (59). Therefore, AVs can enhance their health through the veterinary management of laboratory animals, increasing the likelihood that more animals can be rehomed elsewhere. In a previous study, 16 beagles were rehomed, and the owner responses 4 years later indicated that the majority of the beagles initially experienced separation anxiety, but subsequently, no behavioral issues were reported, and the beagles adapted well to their new homes (75). AVs should recognize the value of extending the lives of sentient beings as a principle that ought to be promoted for the benefit of animals. Moreover, by providing such opportunities to animals, they contribute to fostering a more virtuous and compassionate institution and staff (76).

### 2.4 AV’s responsibility for workplace well-being

So far, we primarily focused on the direction from animals to humans. However, recognizing that animal-human interaction is bidirectional is essential. Building resilience, referring to the ability to cope with stress and adversity and recover effectively, enhances the well-being of both humans and animals (64). We propose extending the responsibility of AVs to include workplace well-being to enhance researchers’ psychological well-being. Recognizing the inevitable stress factors resulting from animal experimentation and developing programs that promote positive workplace cultures through initiatives such as peer counseling and social support is essential. In developed countries, efforts have already been made to develop programs that consider not only animal use but also the emotions of researchers to foster a healthy research environment (77). The program should encompass efforts by researchers to recognize and mitigate stress-inducing factors and psychological and physical risks (78). Additionally, in the context of workplace well-being, fostering open communication among researchers and affirming the scientific value of their work is imperative. This affirmation serves as a reminder that their endeavors improve human health, addressing the negative perceptions of animal experimentation within the public sphere. In a previous study, a survey was conducted with 1,000 employees to identify those experiencing compassion fatigue, and external consultants were engaged for counseling. A well-being program was developed by consolidating various ideas, one of which involved providing employees with dedicated time to distribute special treats for animals. Additionally, to enhance the bond with animals, an animal-dedicated garden was established. Over time, this compassion fatigue program evolved, gaining traction and interest among many employees, ultimately exerting a positive influence on the overall culture of the animal research institution (77).

## 3 Discussion

One of the most significant findings of this study is the substantial impact of Attending Veterinarians (AVs) in animal research facilities on the research capabilities and psychological well-being of researchers. AVs contribute their expertise to enhance the welfare of experimental animals and provide insights to alleviate overall stress and empathy loss among researchers. The research results underscore that AVs play a crucial role in improving the efficiency of research and reducing unnecessary animal use through the proper management of experimental animals, aligning with the principles of the 3Rs (Replacement, Reduction, Refinement). Additionally, by fostering a positive workplace culture, engaging in peer counseling, and providing social support, AVs can enhance the overall work environment in animal research facilities. This, in turn, assists researchers in finding more meaning in their work and mitigates negative perceptions associated with animal research. Therefore, this study addresses the ethical considerations surrounding animal research, emphasizing the need to balance scientific advancement with the welfare of laboratory animals and the psychological well-being of researchers. Veterinarians in developed countries like Australia, the United States, and the United Kingdom experience considerable job-related stress from animal attacks, public perception, and other potential risks (79). Veterinarians significantly experience higher psychological distress compared to the general population (80). Psychological distress is prevalent among veterinarians, physicians, and nurses, especially in intensive care units where end-of-life decisions are made, with 12% reporting experiencing depression (81). This finding underscores the potential exposure to significant stress in their relationships with animals. Therefore, the importance of AV tasks in identifying and caring for animals in pain, particularly in animal experimentation facilities where euthanasia is more frequently performed than in animal or human hospitals, warrants consideration.

In the United States and Canada, the concept of veterinary social workers is emerging to support the mental health of veterinarians performing euthanasia in animal shelters (82), and educational programs for social workers are expanding to include Human-Animal Interaction Contents (82, 83). Therefore, society should consider the mental health of veterinarians in animal shelters and that of researchers in animal experimentation facilities. AV work in such facilities should be recognized as a vital component of social equity, acknowledging the welfare of humans and laboratory animals. The results of this study suggest that the role of AVs can have a significant impact on current practices in animal research facilities. These findings may advocate for some changes in policies and operations within animal research facilities. It is advisable for all facilities conducting animal experiments to employ AVs. Furthermore, continuous education and training for AVs are essential, providing support to stay updated with the latest knowledge in animal management practices. Additionally, AVs should establish transparent and ethical guidelines for rehoming programs and euthanasia procedures. AVs play a pivotal role in developing programs tailored to the sociability and responsiveness levels of each animal, assisting researchers in their roles. Anticipating that these changes, when applied to the policies and operations of animal research institutions, will foster a more ethical environment, providing researchers with more positive experiences and enhancing research capabilities.

However, our study has several limitations. Firstly, further research is needed to explore how the role of AVs varies depending on the species of experimental animals and the characteristics of research facilities. Investigating the impact of AVs on various animal species would be beneficial in exploring optimal welfare and management practices for specific species. Secondly, there is a need for research considering cultural and social variables that influence interactions between AVs and researchers. While the role of AVs is trending toward international standardization, the unique cultural and social atmospheres in each country may differ. Therefore, AVs in each country should find the best practices within their social context through research and regular consultations. Lastly, additional research is needed to statistically verify the level of compassion fatigue among researchers at animal research institutions according to the presence or absence of AV.

Despite these limitations, the findings of this study can contribute to shaping and transforming societal perspectives on animal research, veterinary practices, and animal welfare. The role of AVs exerts a significant influence in these domains, with expectations for contributing to the future development of socially acceptable and advanced research cultures. Emphasizing that the role of AVs goes beyond the responsibility for animal health, impacting the overall quality and welfare of the environment, including the psychological well-being of researchers. Additionally, within the context of veterinary education, the integration of modern technological devices has become a noteworthy topic in discussions, with the utilization of social media platforms being particularly noteworthy (84). Referencing recent research is crucial in exploring avenues to strengthen data collection through such technologies and provide advanced knowledge to future veterinarians (84). In the future, strategies for enhancing education on AVs should continue to evolve by leveraging the various features and capabilities offered by social media platforms. Exploring ways to actively utilize social media in veterinary education through collaboration with AVs from different institutions is essential, raising awareness of the demands and challenges faced by the community. Therefore, highlighting the importance of future research to evaluate the long-term educational effects and understand the practical applications in various learning environments. Efforts to improve education to foster such technological changes are crucial. A study indicated that the Flipped Classroom and Peer-Assisted Learning (FC/PAL) approach significantly contributed to improving students’ achievement (85). This outcome suggests the ongoing need to explore and enhance strategies focusing on interaction and collaboration for AV education.

## 4 Conclusion

This study explored the diverse impacts of attending veterinarians (AVs) in animal research on animal welfare, researchers’ psychological well-being, and the overall research environment. The study emphasize that AVs can enhance the efficiency of research by improving the welfare of experimental animals and reduce animal usage by emphasizing the 3R principles. Additionally, AVs can contribute to improving the workplace environment for researchers through fostering a positive workplace culture and peer counseling, as well as contributing to the meaningfulness of animal research and alleviating negative perceptions.

In the future, it is anticipated that strengthening the education and training of AVs will contribute to maintaining up-to-date knowledge in animal management while playing a crucial role in fostering socially acceptable and advanced research cultures. The employment of AVs in all animal research institutions, along with efforts to enhance education and training, and the establishment of transparent and ethical guidelines have been identified as necessary. Overcoming the limitations of the research, there is a need for additional studies to investigate how the diverse roles of AVs may vary based on the species of experimental animals and the characteristics of research facilities. Research considering variables that influence interactions between AVs and researchers in different national and cultural contexts is also warranted.

These research endeavors are expected to lead to positive changes that enhance the ethical aspects of animal research and the psychological well-being of researchers.

## Data availability statement

The original contributions presented in the study are included in the article/supplementary material, further inquiries can be directed to the corresponding authors.

## Author contributions

JL: Investigation, Writing – review & editing. G-HL: Conceptualization, Investigation, Writing – original draft. KK: Supervision, Writing – review & editing.
